# Relationship between sociodemographic, clinical, and laboratory characteristics and severity of COVID-19 in pediatric patients

**DOI:** 10.1371/journal.pone.0283037

**Published:** 2024-05-07

**Authors:** Cristian Roca, Adriana Asturizaga, Nelson Villca, Ramiro Cabrera, Raul Copana-Olmos, Vladimir Aguilera-Avendano, Claudia Estrada-Villarroel, Mariel Andrea Forest-Yepez, Marcia Torrez-Santos, Adela Felipa Magne-Calle, Maria Ofelia Foronda-Rios, Liz Malena Pena-Helguero, Monica Montalvo, Delina Torrez, Mirna Toco, Miguel Cespedes, Ingrid Davalos, Natalie M. Bowman, COVID19 Research Group in Bolivia

**Affiliations:** 1 Department of Microbiology and Immunology, University of North Carolina at Chapel Hill, Chapel Hill, NC, United States of America; 2 Pediatric Pneumology Department, Caja de Salud de la Banca Privada, La Paz, Bolivia; 3 Pediatric Pneumology Department, HODE Materno Infantil Hospital CNS, La Paz, Bolivia; 4 Pediatric Pneumology Department, Mario Ortiz Children’s Hospital, Santa Cruz, Bolivia; 5 Covid19 Intensive Care Unit, Manuel A. Villarroel Children’s Hospital, Cochabamba, Bolivia; 6 Maternal Infant Department, Medicine Faculty San Simon University, Cochabamba, Bolivia; 7 Pediatric Infectology Department, Ovidio Aliaga Uria Children’s Hospital, La Paz, Bolivia; 8 Pediatric Intensive Care Unit, Materno Infantil Hospital CNS, La Paz, Bolivia; 9 Pediatric Department, North Hospital, El Alto, La Paz, Bolivia; 10 Education and Research Department, Reidun Roine Materno Infantile Hospital, Riberalta, Bolivia; 11 Pediatric Intensive Care Department, Materno Infantil Hospital CNS, Santa Cruz, Bolivia; 12 Department of Pediatrics, Hospital Cooperación Corea, Oruro, Bolivia; 13 Pediatric Department, Bolivian Japanese Hospital Materno Infantil, Trinidad, Beni, Bolivia; 14 Pediatric Intensive Care Department, North Hospital, El Alto, La Paz, Bolivia; 15 Division of Infectious Diseases, Department of Medicine, University of North Carolina, Chapel Hill, NC, United States of America; Stellenbosch University Faculty of Medicine and Health Sciences, SOUTH AFRICA

## Abstract

COVID-19 affects children less seriously than adults; however, severe cases and deaths are documented. This study objective is to determine socio-demographic, clinical and laboratory indicators associated with severe pediatric COVID-19 and mortality at hospital entrance. A multicenter, retrospective, cross-sectional study was performed in 13 tertiary hospitals in Bolivia. Clinical records were collected retrospectively from patients less than 18 years of age and positive for SARS-CoV-2 infection. All variables were measured at hospital entrance; outcomes of interest were ICU admission and death. A score for disease severity was developed using a logistic regression model. 209 patients were included in the analysis. By the end of the study, 43 (20.6%) of children were admitted to the Intensive care unit (ICU), and 17 (8.1%) died. Five indicators were independently predictive of COVID-19 severity: age below 10 years OR: 3.3 (CI95%: 1.1–10.4), days with symptoms to medical care OR: 2.8 (CI95%: 1.2–6.5), breathing difficulty OR: 3.4 (CI95%: 1.4–8.2), vomiting OR: 3.3 (CI95%: 1.4–7.4), cutaneous lesions OR: 5.6 (CI95%: 1.9–16.6). Presence of three or more of these risk factors at hospital entrance predicted severe disease in COVID-19 positive children. Age, presence of underlying illness, male sex, breathing difficulty, and dehydration were predictive of death in COVID-19 children. Our study identifies several predictors of severe pediatric COVID-19 and death. Incorporating these predictors, we developed a tool that clinicians can use to identify children at high risk of severe COVID-19 in limited-resource settings.

## Introduction

In December 2019, an outbreak of respiratory illness was reported in Wuhan (Hubei province, China) caused by a coronavirus now called Severe Acute Respiratory Syndrome Coronavirus 2 (SARS-CoV-2) that causes coronavirus disease 2019 (COVID-19) [1]. By August 2021, the SARS-CoV-2 pandemic had caused more than 4 million deaths worldwide [2]. Although most deaths occurred in people over 65 years of age and individuals with underlying diseases, people from all ages have been affected by this pandemic [3, 4].

Pediatric cases of COVID-19 are frequently asymptomatic or mild [5], however, they represent approximately 2.4% of all severe cases and comprise atypical presentations such as Multisystemic Inflammatory Syndrome (MIS-C) and septic shock syndrome [6, 7]. Furthermore, hematological alterations like anemia and hypercoagulability are also observed in severe pediatric and adult cases [8, 9].

In many countries, the first wave of COVID-19 cases resulted in rapid saturation of hospital beds [10]; for this reason, active case surveillance and diagnosis has become a priority [11]. Unfortunately, in pediatric settings predominantly symptomatic and severely ill children are diagnosed due to the need for hospital care [12]. Moreover, at admission to the hospital, it is not clear which children are going to develop severe COVID-19. Few reports have explored early clinical manifestations of pediatric COVID-19 cases [13], and indicators of severity in low- and middle-income settings remain to be established. This study’s aim is to determine the main socio-demographic, clinical and laboratory indicators associated with severe pediatric COVID-19 at hospital entrance as an early tool to predict severity and mortality of COVID-19 in children.

## Materials and methods

A multicenter, retrospective study was performed in 3 tertiary pediatric hospitals and 10 general hospitals in 5 departments of Bolivia. In Bolivia, the first COVID-19 case was reported on March 10, 2020 [14]. Data from all COVID-19 pediatric cases admitted to the 13 study sites in April through October 2020 (first wave) was abstracted by pulmonologists and pediatricians of each hospital and independently reviewed by peer physicians at same study centers. Inclusion criteria included age less than 18 years and SARS-CoV2 infection diagnosed by RT-PCR, rapid antigen test, or rapid antibody test plus clinical suspicion of COVID-19 or need for hospital care. Patients whose records were missing more than 20% of study measures were excluded.

All information was collected retrospectively from clinical records including socio-demographic, clinical, and laboratory measures at hospital entrance. Status of the patient at the end of study (October 2020) was also recovered to determine the need for intensive care and death. High altitude sites were defined as those located in cities above 2500 meters above sea level (masl) [La Paz (3650 masl), Cochabamba (2558 masl) and Oruro (3709 masl)]; while low altitude (lowlands) were those below 2500 masl [Santa Cruz (416 m masl) and Beni (155 masl)]. Based on the level of care needed and clinical status at the end of the study (October 2020), individuals were classified as hospitalized in the acute care or intensive care unit (ICU) (COVID-19 severity), and as alive (at hospital discharge or in the hospital at the end of October 2020) or deceased (mortality).

### Ethical considerations

This study was approved by the Institutional review board of the Christian University of Bolivia (FWA00028928). The study was considered no risk for the participants since only the participants’ data were recovered. All personal identifiers were eliminated before data analysis. Requirement of individual informed consent was waived by the IRB due retrospective nature of the study and the absence of personal identifiers in the data.

### Statistical analysis

Chi-square test, Fisher’s exact test, Mann-Whitney or the Kruskal Wallis test were used to estimate association between the predictors and COVID-19 severity or mortality. Logistic modelling was carried out to identify most significant indicators to construct a COVID-19 severity score. First, all variables significantly associated with ICU admission were considered for the model. Log-likelihood ratio tests were used to identify variables that significantly contributes to the final model. To facilitate interpretation, variables “age” and “days from symptoms onset until medical attention” were categorized as <10/≥10 years old, and ≤3/>3 days respectively. Supplementary analyses were performed to estimate association between geographic altitude and severity of COVID-19 disease in children. All statistical analyses were performed using the statistical software STATA v.16.1. (College Station, TX). Figure was performed using GraphPad Prism V.9.0 (GraphPad Software Inc., CA).

## Results and discussion

Two hundred nine patients were included in the analysis, of which 166 (79.4%) were hospitalized in acute care and 43 (20.6%) admitted to the ICU. Socio-demographic characteristics, clinical features, and laboratory indicators were evaluated as early indicators of pediatric COVID-19 severity and mortality in children positive to COVID-19 at secondary and tertiary hospitals in 5 regions of Bolivia.

As shown in Table 1, children requiring ICU admission were younger than those managed in the hospitals’ acute care wards (p<0.01). Children from the highlands were less frequently admitted to ICU (OR 0.15, 95% CI 0.06, 0.34) than children in lowlands locations. ICU admission was also less common in children from urban settings than rural areas (OR 0.36; 95%CI 0.17, 0.77). Other socio-demographic factors were not significantly associated with ICU admission. Greater number of days from symptom onset to medical care or to diagnosis was also associated with ICU care (p<0.01).

**Table 1:** Association of socio-demographic characteristics and need for ICU care (N = 209) and in-hospital mortality (N = 159) in Bolivian children hospitalized with SARS-CoV-2 infection.

		Severity of Pediatric COVID-19					Mortality				
		Hospitalized (n = 166)		Intensive Care Unit (n = 43)		p-value	Alive (n = 142)		Deceased (n = 17)		p-value
		n	(%)	n	(%)		n	(%)	n	(%)	
Age (years)*		6.5	(1–11)	4	(0–8)	<0.01	6	(1–10)	4	(0–8)	0.18
Male Gender		98	(59.0)	29	(67.4)	0.32	85	(59.9)	15	(88.2)	0.04
Body Mass Index*		17	(15–19)	17	(14–19)	0.85	17	(15–20)	15	(14–16)	0.04
Urban area		138	(83.6)	28	(65.1)	<0.01	119	(83.8)	12	(70.6)	0.19
Altitude											
	Highlands	100	(60.2)	8	(18.6)	<0.01	80	(56.3)	11	(64.7)	0.51
	Lowlands	66	(39.8)	35	(81.4)	ref.	62	(43.7)	6	(35.3)	ref.
Currently Vaccinated (Influenza)^b^		8	(5.0)	2	(4.8)	0.95	----	----	----	----	----
Parent’s educational level											
	None or Primary	38	(23.9)	15	(36.6)	ref.	33	(24.3)	9	(52.9)	ref.
	Secondary or Higher	121	(76.1)	26	(63.4)	0.10	103	(75.7)	8	(47.1)	0.02
Household monthly income											
	< 2500Bs.	31	(25.4)	12	(34.3)	ref.	31	(29.3)	2	(20.0)	ref.
	2500-50000Bs.	64	(52.5)	16	(45.7)	0.32	54	(50.9)	6	(60.0)	0.52
	>5000Bs.	27	(22.1)	7	(20.0)	0.46	21	(19.8)	2	(20.0)	0.71
Presence of underlying illness		90	(54.2)	22	(51.2)	0.72	68	(47.9)	14	(82.3)	0.01
Days with symptoms to medical care^e^*		3	(1–5)	4	(3–7)	< 0.01	3	(1–5)	4	(2–6)	0.20
Days with symptoms to diagnosis*		3	(1–7)	5	(4–8)	< 0.01	4	(1–7)	4	(1–6)	0.89
* Presented as Median (Interquartil range), p-values obtained by Mann-Whitney test							* Presented as Median (Interquartil range), p-values obtained by Mann-Whitney test				
^a^ P-values and Odds Ratio obtained by unajusted logistic regression Wald test.							^a^ Outcome: Deceased (n = 17) / Discharged or remain hospitalized (n = 142)				
^b^ 6 missing values in Hospitalized group, and 1 missing in ICU							^a^ p-value by unajusted logistic regression Wald test				
^c^ 2 missing values in ICU group, and 7 missing in Hospitalized							^b^ 11 missing values in Discharged/remain hospitalized group				
^d^ Only 122, and 35 participants in Hospitalized, and ICU groups respectively							^c^ 5 missing values in Discharged/remain hospitalized group				
^e^ Only 158, and 42 participants in Hospitalized, and ICU groups respectively							^d^ 6 missing values in Discharged/remain hospitalized group				
^f^ Only 154, 43 participants in Hospitalized, and ICU groups respectively							^e^ 7, 36 missing values in Deceased and Discharged/remain hospitalized groups respectively				
							** No deceased child had a recent influenza vaccine				

Mortality data was available for 159 of the 209 participants, of which 17 (10.7%) died and 142 were either discharged alive from the hospital or remained hospitalized at the study endpoint. Male gender (OR: 5.03; CI95%: 1.11 to 22.84; p = 0.04), decreased body mass index (p = 0.04), lack of parental education (OR: 0.28; CI95%: 0.10 to 0.80; p = 0.02), and presence of underlying illness (OR: 5.08; CI95%: 1.40 to 18.44; p = 0.01) were significantly associated with mortality in COVID-19 infants (Table 1). Children with missing data on mortality did not differ with respect to these variables, though there was a trend towards younger age (p = 0.10) in children with missing mortality data (S1 Table).

Univariate analyses of the relationships between signs and symptoms at hospital entrance and ICU admission and mortality are shown in Table 2. Fever, breathing difficulty, fatigue, diarrhea, vomiting, dehydration, cyanosis, crepitant rales, nasal flaring, and cutaneous lesions were significantly associated with ICU admission; breathing difficulty, fatigue, dehydration, cyanosis, rales, and nasal flaring were significantly associated with mortality in our study population.

**Table 2:** Factors associated with pediatric COVID-19 severity and mortality at tertiary hospitals in Bolivia (N = 209).

Factor	Severity of Pediatric COVID-19						Mortality					
	Hospitalized (n = 166)		ICU (n = 43)		OR (95% CI)	p-value	Alive (n = 142)		Deceased (n = 17)		OR (95% CI)	p-value
	n/N*	(%)	n/N*	(%)			n	(%)	n	(%)		
Self-reported fever	106/164	(64.6)	32/43	(74.4)	1.59 (0.75, 3.39)	0.23	96	(67.6)	9	(52.9)	0.54 (0.19, 1.49)	0.23
Fever at hospital entrance	26/164	(15.8)	14/43	(32.6)	2.56 (1.19, 5.49)	0.02	23	(16.3)	5	(31.2)	2.33 (0.74, 7.35)	0.15
Fatigue	49/164	(29.9)	22/43	(51.2)	2.46 (1.24, 4.88)	0.01	51	(35.9)	11	(64.7)	3.27 (1.14, 9.37)	0.03
Cough	70/165	(42.4)	21/42	(50.0)	1.36 (0.69, 2.68)	0.38	57	(40.4)	11	(64.7)	2.70 (0.94, 7.72)	0.06
Breathing difficulty	48/164	(29.3)	22/43	(51.2)	2.53 (1.27, 5.03)	< 0.01	42	(29.6)	13	(76.5)	7.74 (2.38, 25.11)	< 0.01
Wheezing	21/162	(13.0)	10/43	(23.3)	2.03 (0.87, 4.73)	0.10	20	(14.3)	3	(17.6)	1.28 (0.34, 4.88)	0.10
Cyanosis	10/162	(6.2)	17/43	(39.5)	9.94 (4.10, 24.08)	< 0.01	10	(7.1)	13	(76.5)	42.25 (11.60, 153.80)	< 0.01
Crepitant rales	44/162	(27.2)	22/43	(51.2)	2.81 (1.41, 5.61)	< 0.01	37	(26.4)	11	(64.7)	5.10 (1.76, 14.78)	< 0.01
Pharyngeal pain	16/164	(9.8)	8/43	(18.6)	2.11 (0.84, 5.33)	0.11	16	(11.3)	4	(23.5)	2.42 (0.70, 8.33)	0.16
Pharyngeal erythema	34/162	(21.0)	12/43	(27.9)	1.46 (0.68, 3.13)	0.33	31	(22.1)	5	(29.4)	1.46 (0.48, 4.48)	0.50
Rhinorrea	26/164	(15.9)	7/43	(16.3)	1.03 (0.42, 2.57)	0.95	23	(16.2)	1	(5.9)	0.32 (0.04, 2.56)	0.28
Nasal Congestion	20/164	(12.2)	6/43	(13.9)	1.17 (0.44, 3.11)	0.76	20	(14.1)	1	(5.9)	0.38 (0.05, 3.03)	0.36
Nasal Flaring	12/162	(7.4)	12/43	(27.9)	4.84 (1.99, 11.77)	< 0.01	12	(8.6)	11	(64.7)	19.55 (6.14, 62.22)	< 0.01
Diarrhea	24/165	(14.6)	16/43	(37.2)	3.48 (1.64, 7.40)	< 0.01	26	(18.3)	2	(11.8)	0.59 (0.13, 2.76)	0.51
Vomiting	46/164	(28.1)	24/43	(55.8)	3.24 (1.62, 6.47)	< 0.01	47	(33.1)	4	(23.5)	0.62 (0.19, 2.01)	0.43
Dehydration	38/162	(23.5)	21/43	(48.8)	3.11 (1.54, 6.27)	< 0.01	33	(23.6)	9	(52.9)	3.65 (1.30, 10.21)	0.01
Cutaneous lesions	11/162	(6.8)	12/43	(27.9)	5.31 (2.15, 13.13)	< 0.01	18	(12.9)	1	(5.9)	0.42 (0.05, 3.39)	0.42
Epileptic movements	6/162	(3.7)	5/43	(11.6)	3.42 (0.99, 11.81)	0.05	4	(2.9)	1	(5.9)	2.12 (0.22, 20.19)	0.51

* n = symptomatic individuals; N = Total population in the group.

^a^ P-values and Odds Ratio obtained by unajusted logistic regression Wald test.

Laboratory indicators at hospital entrance were analyzed (Table 3) to explore association with disease severity. Respiratory frequency (p<0.01), white blood cells (WBC) count(p<0.01), AST (p = 0.03) and ALT (p<0.01) were associated with severe pediatric COVID-19. COVID-19 Mortality analyses (Table 3) showed that respiratory frequency (p = 0.04), oxygen saturation (p<0.01), WBC (p<0.01), and ALT (p = 0.03), were the only indicators statistically associated with mortality in COVID-19 positive infants. In addition, oxygen saturation, hemoglobin, WBC and C-reactive protein were statistically different in highland locations compared to lowlands locations in COVID-19 positive children (S2 Table).

**Table 3:** Indicators associated with pediatric COVID-19 severity in tertiary hospitals in Bolivia.

Factor	Severity of Pediatric COVID-19							Mortality						
	Hospitalized			Intensive Care Unit			p-value^a^	Alive (n = 142)			Deceased (n = 17)			p-value^a^
	n	Median	(IQR)	n	Median	(IQR)		n	Median	(IQR)	n	Median	(IQR)	
Respiratory rate (/min)	165	28	(24–34)	39	35	(28–40)	< 0.01	140	28	(24–34)	15	43	(20–52)	0.04
Oxygen Saturation (%)	164	90.0	(82.0–96.0)	42	93.0	(86.0–98.0)	0.21	141	91.0	(83.0–96.0)	16	81.0	(73.5–87.0)	< 0.01
Hemoglobin (g/dL)	157	11.8	(9.6–13.9)	42	10.0	(9.5–12.8)	0.06	136	11.8	(9.7–13.8)	16	11.1	(8.0–11.8)	0.99
White blood cells (x10^3^ /mm^3^)	156	10.0	(6.8–16.2)	42	14.6	(10.0–18.6)	< 0.01	134	10.4	(6.9–15.8)	15	11.0	(5.2–14.7)	< 0.01
Lymphocytes (%)	129	25.0	(14.0–40.0)	40	20.0	(14.0–41.0)	0.83	114	23.0	(14.0–40.0)	11	24.0	(10.0–45.0)	0.86
Platelets (x10^4^ /mm^3^)	156	27.5	(20.3–36.0)	42	22.1	(13.0–38.5)	0.14	135	26.7	(20.0–35.3)	16	24.6	(13.2–39.2)	0.51
ALT (U/L)	90	30	(18–44)	37	40	(29–84)	< 0.01	86	30	(19–47)	13	39	(34–55)	0.03
AST (U/L)	90	26	(18–44)	37	38	(21–62)	0.03	86	28	(19–45)	13	39	(20–46)	0.39
Serum sodium (mEq/L)	121	137	(134–140)	42	135	(131–139)	0.16	108	136	(133–139)	16	135	(127–141)	0.71
Serum potasium (mEq/L)	121	3.9	(3.5–4.3)	40	3.9	(3.1–4.5)	0.73	107	3.9	(3.5–4.4)	16	4.2	(3.5–5.0)	0.25
Serum C-reactive protein (mg/L)	146	34	(6.0–96.0)	41	40	(6.0–96.0)	0.88	130	24	(6.0–96.0)	16	28	(6.0–96.0)	0.63

^a^ P-values obtained by Mann-Whitney test.

**Fig 1:**
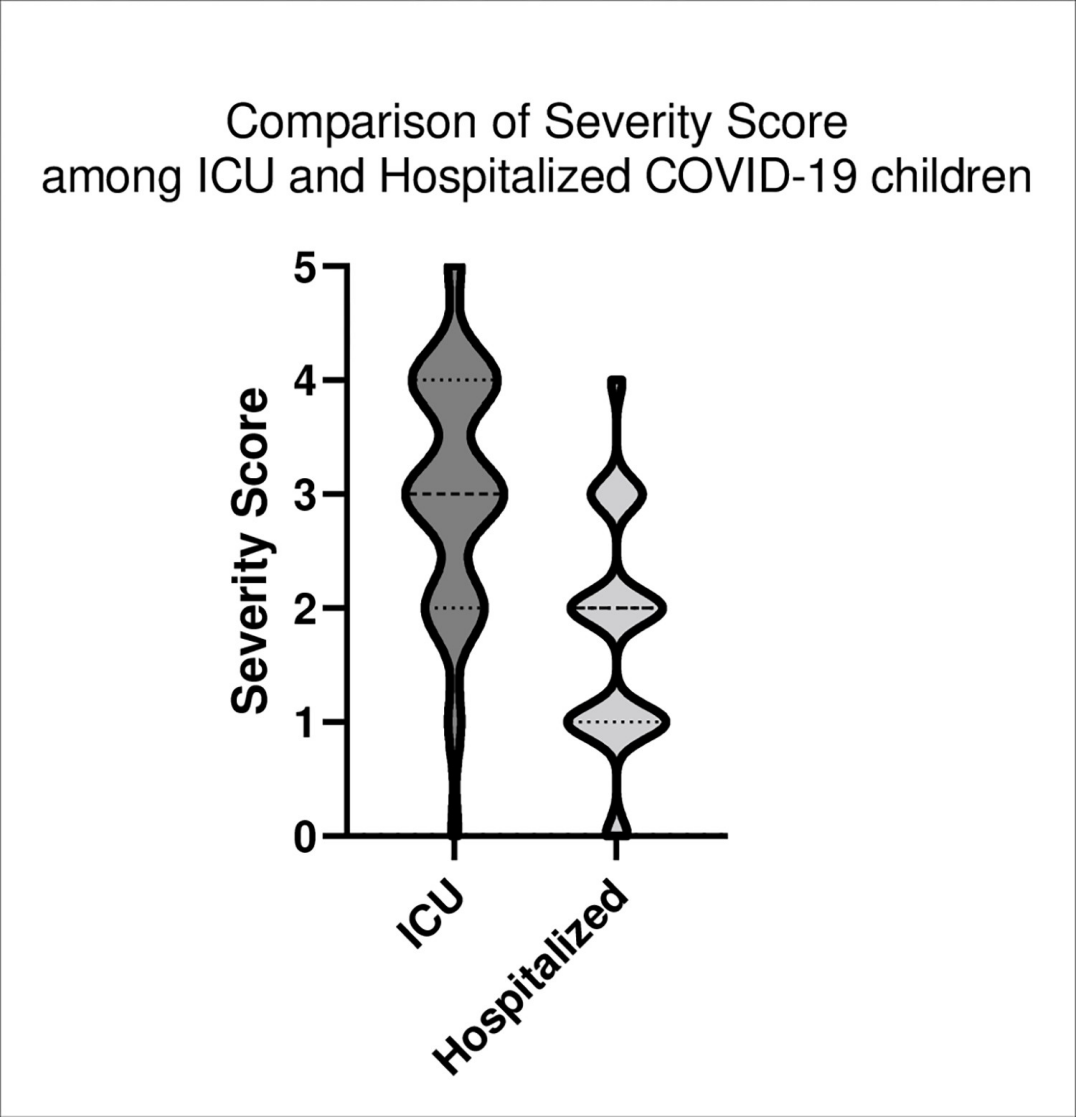
Violin plot showing differences in the distribution of patients with 0 to 5 points in our severity score among ICU admitted vs hospitalized only COVID19-positive patients.

**Fig 2:**
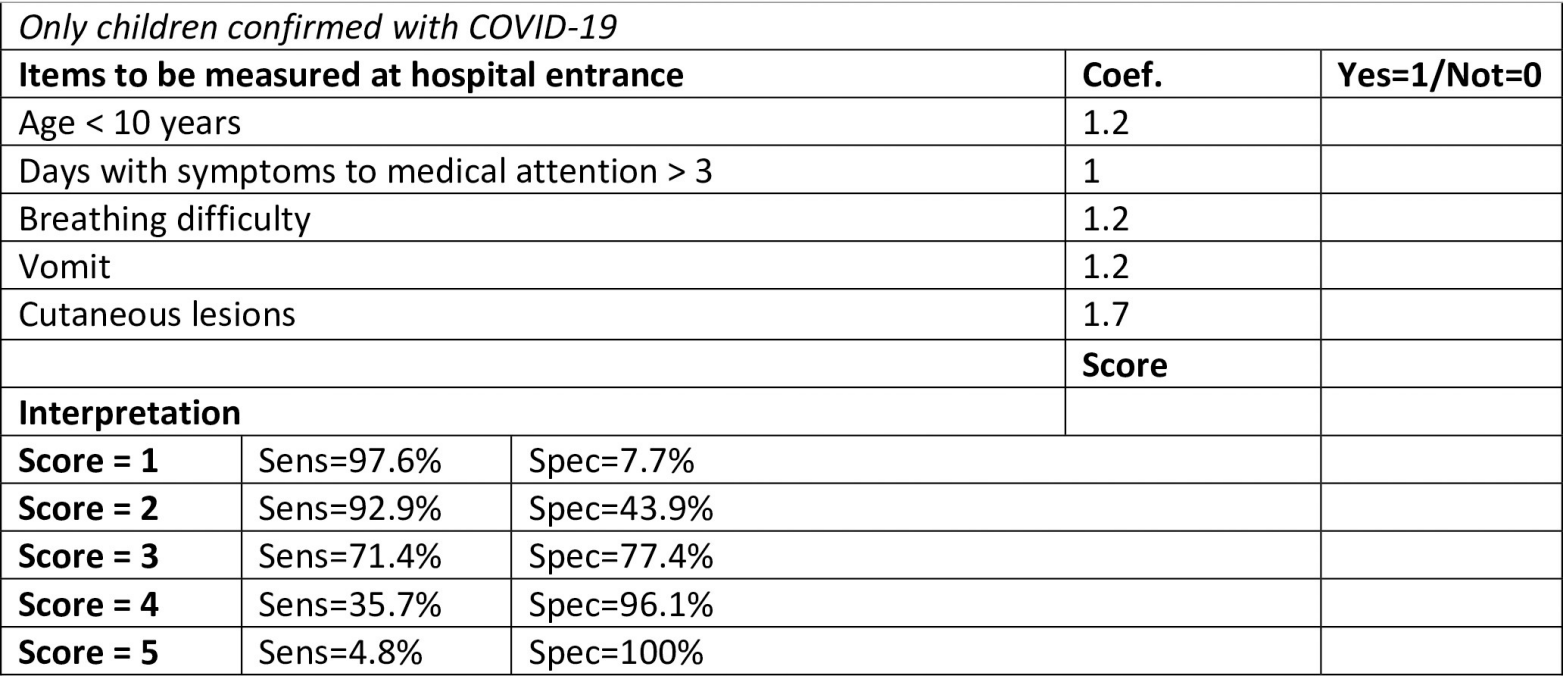
Score to predict severity in pediatric COVID-19. Only children confirmed with COVID-19.

We developed a model using logistic regression to predict severe pediatric COVID-19 requiring ICU admission (Table 4). Five indicators were included in our final model: age below 10 years (OR 3.3; CI95%: 1.1 to 10.4), more than 3 days between symptom onset and medical care (OR: 2.8; CI95%: 1.2 to 6.5), breathing difficulty (OR: 3.4; CI95%: 1.4 to 8.2), vomiting (OR: 3.3; CI95%: 1.4 to 7.4), and cutaneous lesions (OR: 5.6; CI95%: 1.9 to 16.6). Using coefficients from the model, we developed a score (Figs 1 and 2) to estimate the risk of severe pediatric COVID-19 early at hospital entrance. Compared with score 0, score = 3 predicts moderate risk of severe disease (OR: 6.2; CI95%: 0.7 to 52.4); score = 4 predicts high risk of severe disease (OR: 26.0; CI95%: 2.7 to 248.6), and score = 5 predicts extreme risk of severe disease (all children with 5 items were admitted to ICU), with a sensitivity of 71.4%, 35.7%, 4.8% and specificity of 77.4%, 96.1% and 100%, respectively.

**Table 4:** Multivariable modeling of pediatric COVID-19 severity (N = 197).

	Coef.	OR (95%CI)	p-value
Age < 10 years	1.2	3.3 (1.1–10.4)	0.04
Days with symptoms to medical care* > 3	1.0	2.8 (1.2–6.5)	0.02
Breathing difficulty	1.2	3.4 (1.4–8.2)	<0.01
Vomiting	1.2	3.3 (1.4–7.4)	<0.01
Cutaneous lesions	1.7	5.6 (1.9–16.6)	<0.01

† Outcome: Intensive care unit vs Hospitalized.

Statistical Model. Adjusting for all predictors in this table.

P-value obtained by multiple logistic regression Wald test.

* Categorized as ≤3 and >3 days.

A logistic model of indicators associated with mortality was also constructed (Table 5). Presence of underlying illness (p<0.01), breathing difficulty (p = 0.04), dehydration (p = 0.01) were predictors of COVID-19 mortality in children, adjusted for age and sex. sample size precluded development of a predictive model for mortality due to imprecise estimations.

**Table 5:** Multivariable modeling of pediatric COVID-19 mortality (N = 159).

Variables	Statistical Model	
	OR (95%CI)	p-value
Age	0.90 (0.80, 1.02)	0.11
Gender (male)	5.08 (0.97, 26.62)	0.05
Presence of Underlying Illness	8.31 (1.74, 39.51)	<0.01
Breathing difficulty	3.95 (1.09, 14.31)	0.04
Dehydration	5.28 (1.44, 19.19)	0.01

† Outcome: Deceased vs Discharged/remain hospitalized.

Statistical Model. Adjusting for all predictors in this table.

P-value obtained by multiple logistic regression Wald test.

Our study identified sociodemographic, clinical and laboratory predictors of severe pediatric COVID-19 and mortality in Bolivia during the first wave of the SARS-CoV-2 pandemic. This is one of the first large studies of pediatric COVID-19 patients from a resource-limited setting, where risk factors for severe COVID-19 in children may differ from those in high-income countries due to higher prevalence of undernutrition, parasitic infections, and other diseases of poverty as well as decreased access to medical care. We also captured a geographically diverse population, allowing us to explore the effect of high altitude on COVID-19 severity.

The first wave of the SARS-CoV-2 pandemic in Bolivia (April-October 2020) caused more than 140,000 confirmed cases, of which approximately 3.5% were children under 15 years of age [2, 15, 16]. Although most children experience mild or asymptomatic infection, some require hospital attention due to severe forms of COVID-19 that can cause death [17]. In our study, we observed that more severe forms of COVID-19 were present in younger children and that male children had higher mortality than females. Other recent reports have shown that hospitalization due to COVID-19 is more frequent at age below 1 year [18], and severity of the disease is increased in male individuals [19].

We also observed that time from symptom onset to medical attention and diagnosis were associated with disease severity (Table 1). We hypothesize that delay in seeking medical attention for children increases risk of severe COVID-19. Parental education may play a role in deciding to explore alternative home care instead of seeking medical attention; higher parental education may also be a marker of wealth and greater access to medical treatment. The presence of underlying illness was an independent risk factor for mortality but not ICU admission in our patient population (Tables 4 and 5). As our study population was pulled from tertiary care centers, some of the children in the study may have died of the underlying illnesses treated at such hospitals (for example, cancers) rather than COVID-19.

We found that breathing difficulty and gastrointestinal manifestations (vomiting and diarrhea) were useful indicators of disease severity, corroborating previous reports [20, 21]. Gastrointestinal manifestations were collinear, so we choose vomiting for our model based on best model obtained by log likelihood ratio estimations. In our study, fatigue, nasal flaring, and cyanosis were also associated with COVID-19 severity (Table 2); however, these factors had statistical collinearity between each other and with breathing difficulty so could not be included in the multivariable models. Breathing difficulty was chosen for our statistical model due to better precision with this variable (Tables 3 and 4). Cutaneous lesions were also predictive of severe COVID-19 as has been previously reported [22].

Respiratory frequency, WBC, ALT, and AST were all increased in critically ill children (Table 3). Remaining laboratory factors were not associated with COVID-19 severity or mortality. Although higher C-reactive protein has been previously associated with COVID-19 mortality and has been previously shown as an useful tool to estimate severe manifestations of COVID-19 [23, 24], in our study we did not observe those associations, which can be explained by methodological and sampling differences between studies.

At hospital entrance, it is difficult to predict which SARS-CoV-2-infected children will develop severe manifestations of the disease, and analysis of all factors associated with this in hospital settings is not feasible. For this reason, we developed a score (Fig 2) based on a statistical model of readily available clinical and demographic indicators associated with COVID-19 severity (Table 4). This score can be used in children under 15 years of age at hospital entrance to predict the risk of moderate or severe disease, identifying children who may warrant more intensive observation or earlier treatment.

Bolivia’s large altitude gradient allowed us to examine differences between outcomes and covariates in children living in high and low altitude settings. Millions of Bolivians live at altitudes that cause chronic hypoxia. It is unknown if physiological adaptations to residence in high altitude would be advantageous in the setting of SARS-CoV-2 infection, or if lower baseline oxygen saturation would predispose to more severe disease. As shown in S2 Table, we found expected differences in hemoglobin, WBC and oxygen saturation in highlands compared to lowlands. Those differences are not pathological and are normal adaptations to life at high altitude [25, 26]. Children residing in high altitude settings were actually less likely to require ICU level of care, though there was no significant association between altitude and mortality. Unfortunately, our study design did not allow us to determine if the observed differences in outcomes were due to altitude-related physiological adaptations or to unmeasured confounders. Furthermore, cases were captured at different epidemic times in lowlands and highlands (number of total cases were higher in lowlands than highlands), which could have contributed to the apparent greater number of severe cases in lowlands in a moment with greater global cases in the region with overwhelmed healthcare facilities.

Our study had several limitations. First, as a retrospective, tertiary hospital-based study, we cannot generalize our findings to children who do not seek medical care, and the participating hospitals were the most complex in each region, so our study population, with a high prevalence of comorbid medical conditions, may not be reflective of the general population. Additionally, we were unable to capture mortality or rehospitalization data after discharge from the hospital. We were not able to develop a predictive model for mortality because missing data produced imprecise estimation. Finally, because this was a retrospective study, we were unable to validate our score prospectively; future studies should test its clinical utility in other populations. SARS-CoV-2 viral variants were not determined, however, during the study period (first wave of cases in Bolivia) only alpha SARS-CoV-2 were detected in Bolivia.

## Conclusions

In this study, we describe risk factors for ICU care and mortality in a large, geographically diverse cohort of children in the low-income country of Bolivia. We did not find a significant interaction between altitude and disease severity in our study. We did identify several clinical predictors of need for ICU level of care and of death and were able to incorporate these into a simple score that clinicians can use to identify children at high risk of severe COVID-19 at hospital admission. Further studies are needed to evaluate altitude’s contribution to COVID-19 pathogenesis, treatment response in children, and inflammatory patterns in pediatric COVID-19 at different stages of the disease.

## COVID19 Research Group in Bolivia

Mauricio Mendoza Perales, Brenda Vasquez, Andrea Mancilla, Gabriela Castro, Teresa Antelo Melgar, Juan Antonio Bravo Serrano, Pablo Mattos Navarro, Jenny Loayza Laura, Jorge Salazar Fuentes.

## Supporting information

S1 Checklist Inclusivity in global research questionnaire. (DOCX)

S1 Data (XLS)

S1 Table Comparison of mortality indicators in children with missing versus complete data of mortality outcome. (DOCX)

S2 Table Factors associated with altitude in pediatric COVID-19 patients. (DOCX)
