# Supplementary material for: Relationship between sociodemographic, clinical, and laboratory characteristics and severity of COVID-19 in pediatric patients
Source: PLoS One. 2024 May 7;19(5):e0283037. doi: 10.1371/journal.pone.0283037 (PMC11075884; doi:10.1371/journal.pone.0283037)
Supplement: S1 Table — (DOCX) [file pone.0283037.s003.docx]

| **Table S1. Comparison of mortality indicators in children with missing versus complete data of mortality outcome** | | | | | |
| --- | --- | --- | --- | --- | --- |
| Variables | Missing mortality data | | Complete data | | p-value |
|  | N=50 | (%) | N=159 | (%) |  |
| Age (median, IQR) | 2.5 | (1-9) | 6.0 | (1-10) | 0.10 |
| Male sex | 27 | (54.0) | 100 | (62.9) | 0.26 |
| Presence of underlying illness | 30 | (60.0) | 82 | (51.6) | 0.30 |
| Breathing difficulty | 15 | (31.2) | 55 | (34.6) | 0.67 |
| Dehydration | 17 | (35.4) | 42 | (26.7) | 0.25 |
| IQR: interquartile range | | | | | |
| p-values obtained by chi-square or Mann-Whitney test | | | | | |
