# Supplementary material for: Relationship between sociodemographic, clinical, and laboratory characteristics and severity of COVID-19 in pediatric patients
Source: PLoS One. 2024 May 7;19(5):e0283037. doi: 10.1371/journal.pone.0283037 (PMC11075884; doi:10.1371/journal.pone.0283037)
Supplement: S2 Table — (DOCX) [file pone.0283037.s004.docx]

| **Table S2. Factors associated with altitude in pediatric COVID-19 patients** | | | | | | | |
| --- | --- | --- | --- | --- | --- | --- | --- |
|  | Lowlands (n=101) | | | Highlands (n=108) | | | p-value^a^ |
|  | n | Median | (IQR) | n | Median | (IQR) |  |
| Respiratory rate (/min) | 97 | 30 | (25 - 40) | 105 | 28 | (24 - 32) | 0.14 |
| Oxygen Saturation (%) | 99 | 97.0 | (92.0 - 98.0) | 107 | 85.0 | (80.0 - 90.0) | < 0.01 |
| Hemoglobin (g/dL) | 96 | 10.4 | (9.2 - 11.8) | 103 | 13.2 | (10.9 - 15.0) | < 0.01 |
| White blood cells (x10^3^ /mm^3^) | 97 | 13.1 | (9.4 - 18.0) | 101 | 8.9 | (5.9 - 13.0) | < 0.01 |
| Lymphocytes (%) | 90 | 23.5 | (14.0 - 42.0) | 79 | 24.0 | (10.0 - 38.0) | 0.34 |
| Platelets (x10^4^ /mm^3^) | 96 | 27.7 | (17.6 - 38.3) | 102 | 25.7 | (18.6 - 32.4) | 0.22 |
| ALT (U/L) | 66 | 33 | (21 - 50) | 61 | 32 | (22 - 48) | 0.61 |
| AST (U/L) | 66 | 28 | (18 - 50) | 61 | 30 | (20 - 46) | 0.62 |
| Serum sodium (mEq/L) | 74 | 136 | (131 - 139) | 89 | 136 | (133 - 140) | 0.38 |
| Serum potasium (mEq/L) | 72 | 3.9 | (3.2 - 4.3) | 89 | 4.0 | (3.5 - 4.6) | 0.11 |
| Serum C-reactive protein (mg/L) | 93 | 24 | (6.0 - 96.0) | 94 | 55 | (16.0 - 128.0) | < 0.01 |
| ^a^ P-values obtained by Mann-Whitney test | | | | | | | |
